# Comparison of Circular and Rectangular-Shaped Electrodes for Electrical Impedance Myography Measurements on Human Upper Arms

**DOI:** 10.3390/mi14061179

**Published:** 2023-05-31

**Authors:** Mohammad A. Ahad, Somen Baidya, Md. Nurul Tarek

**Affiliations:** Department of Electrical and Computer Engineering, Georgia Southern University, Statesboro, GA 30458, USA; someneee06@gmail.com (S.B.); mtare002@fiu.edu (M.N.T.)

**Keywords:** circular electrode, electrode configuration, electrical impedance myography, finite element method

## Abstract

Electrical Impedance Myography (EIM) is a painless, noninvasive approach for assessing muscle conditions through the application of a high-frequency, low-intensity current to the muscle region of interest. However, besides muscle properties, EIM measurements vary significantly with changes in some other anatomical properties such as subcutaneous skin-fat (SF) thickness and muscle girth, as well as non-anatomical factors, such as ambient temperature, electrode shape, inter-electrode distance, etc. This study has been conducted to compare the effects of different electrode shapes in EIM experiments, and to propose an acceptable configuration that is less dependent on factors other than the cellular properties of the muscle. Initially, a finite element model with two different kinds of electrode shapes, namely, rectangular (the conventional shape) and circular (the proposed shape) was designed for a subcutaneous fat thickness ranging from 5 mm to 25 mm. The study concludes, based on the FEM study, that replacing the conventional electrodes with our proposed electrodes can decrease the variation in EIM parameters due to changes in skin-fat thickness by 31.92%. EIM experiments on human subjects with these two kinds of electrode shapes validate our finite element simulation results, and show that circular electrodes can improve EIM effectiveness significantly, irrespective of muscle shape variation.

## 1. Introduction

Bioelectricity became a topic of interest 150 years ago. The production of electricity in muscles and nerves was an important discovery as it has contributed to understanding the underlying cellular construction of muscles and nerves, as well as motor units. Later, Electromyography (EMG) and nerve conduction studies (NCS) were invented, allowing the measurement of action potentials of fibers and motor units. Since then, EMG and NCS have been the two major standard clinical tools used by physicians and technicians for the assessment of muscle and nerve health [1]. EMG provides useful information about tissue composition and its architecture. Needle EMG is the most common neurological test administered in clinical settings for the detection of neuromuscular disorders [2]. A needle is inserted into the region of interest of the muscle and a small excitation pulse is applied in the vicinity of the muscle; the subsequent response of the muscle while contracting is captured through the needle for assessment. Needle EMG is extremely sensitive to the placement of the needle in the muscle and its measurements depend heavily on the expertise of the clinician or technician. The reproducibility of EMG measurements is also very low. Moreover, because of its invasive nature, needle EMG is painful and not suitable for children. On the other hand, noninvasive surface EMG (sEMG) has been extensively researched atacademics; however, its clinical acceptance is still low as the information gathered through sEMG is not clinically relevant in most cases [3]. 

Although bioimpedance measurements were seldom used for characterizing biological tissues in the early 20th century, they gained more attention in the early 1950s when full-body impedance measurements were introduced for nutritional purposes. Bioelectrical Impedance Analysis (BIS) is now a standard medical assessment tool for nutritionists, for the measurement of whole-body water content and body fat analysis [4]. Only in the early 2000s was the idea of electrical impedance myography (EIM) introduced, in which, instead of full-body impedance analysis, localized muscle impedance is measured in an attempt to detect neuromuscular diseases. The technique was applied to mild neuromuscular conditions, such as post-surgery muscle disuse, and to severe diseases, such as amyotrophic lateral sclerosis (ALS), in order to monitor the progression or recession of diseases [5]. Although EIM is in the early phase of refinement, encouraging results have been found through this EIM method in the evaluation of neuromuscular diseases. Along with EMG and EIM, some other techniques, such as ultrasound and MRI, have also gained attention for the application of neuromuscular disease evaluation. For example, ultrasonography can be applied to a specific muscle region and the image obtained is then reconstructed to evaluate the underlying muscle condition. Though noninvasive, this technique is more expensive than EIM, the reconstructed image does not provide all the information needed to evaluate the muscle or nerve condition, and extensive image-processing experience is required in order to analyze the resultant image. While MRI may be used for tumor detection and to find neuromuscular disorders, in particular nerve lesions, it is very expensive, takes a long time and is not suitable for children even though the system is noninvasive and painless. 

Bioimpedance measures the electrical responses of biological tissues, and unveils the underlying electro-physiological properties, in particular the conductance and the permittivity, in terms of resistance and reactance. When measured from the skin, bioimpedance incorporates skin, fat, muscle tissues and bones in the region of the skeletal muscle where electrodes are placed. Applied current thus flows through the skin, fat and muscle tissues, though most of the current flows through the muscle, since skin and fat have tenfold less conductance than that of muscle tissues. At the cellular level, muscle tissue is most commonly treated as having extracellular fluid that can be represented as an extracellular resistor that is parallel to the combination of intercellular fluid, represented as an intercellular resistor, and the cell membrane, represented as a capacitor. This three-element model is frequently used to describe the electrical behavior of muscle tissue. A five-element model is also common, in which an extra resistor and a capacitor, connected together in series, are parallel to the three-element model, in order to incorporate the current through organelles [6]. 

Neuromuscular diseases alter the cellular composition of muscle tissues, and thus affect the cellular electrical properties of conductance and permittivity. This was the major motivational aspect of the idea of EIM. Since EIM measures impedance on the surface of a localized muscle, any change in conductance and permittivity will change the measured impedance values, which are in the range of a few ohms. For example, studies in [7] show that the average reactance for the leg muscles of healthy rats was 22.1 (±2.1) ohms, and after a sciatic nerve injury the average reactance was 16.6 (±1.19) ohms. 

Neuromuscular disease affects the nerves that control voluntary muscles. This phenomenon is reflected in the electrical properties of muscles. EIM is an impedance-based tetra-polar technique that can detect the changes in muscle properties during disuse atrophy [5,8,9] through the application of Ohm’s law. A four-electrode, as opposed to a two-electrode, impedance measurement is very common while measuring bioimpedance, since the former can protect itself from the electrode polarization. Resistance (R), reactance (X) and phase (θ) are the three major parameters assessed in EIM. Abnormal muscle conditions can be identified by the deviation of EIM parameters from those obtained in normal conditions [10,11]. These deviations are also dependent on a number of other factors. For example, the size and shape of the muscle, and the orientation of muscle fibers both affect the data [12,13]. There are also some nonmuscular anatomical factors which impact the measurements. Subcutaneous fat thickness, choice of electrode and inter-electrode distances are among them. Currently, when EIM is used as a tool in neurophysiological and medical settings, rectangular-shaped electrodes are generally used. In this paper, we plan to formulate an optimum electrode shape for EIM measurements, as for EIM to be used as a clinical tool, the electrode parameters need to be standardized [5,14]. Our goal is to observe the effect of electrode-shape variation in regards to skin-fat variation, and propose one shape that meets the demand.

This study makes use of both simulations and experiments on human subjects. For the analysis of the effects of different electrode shapes, a model of the human upper arm was developed using the finite element method (FEM), which has already been established as an appropriate approach for the analysis of asymmetrical shapes when assessing disease-induced alterations of muscle through EIM. To validate our results, we then performed the experiment on human subjects. 

## 2. Materials and Methods

The outer two electrodes used in EIM experiments are called the excitation electrodes, one of which is the current-injecting voltage source, while the other is the sink, or ground. The inner two electrodes are known as the sense electrodes since they evaluate the potential difference between two points in the muscle region of interest. The three basic parameters that can be assessed easily from EIM experimentation are resistance (R), reactance (X) and phase (θ). The study is conducted over a large range of frequencies. These parameters show different characteristics throughout the frequency domain. Deviation from the normal profile of EIM parameters can occur due to changes in the muscle’s electrical properties during disease progression [15]. The methodology that is implemented in EIM is expressed by Ohm’s Law:

*V* = *IZ* (1)

where *V* is the voltage, *I* is the current flow and *Z* is the impedance, which is explored in EIM for disease detection. The measured complex impedance from the experiment can be written as:

*Z* = *R* + *jX* (2)

So the complex admittance becomes:

*Y* = *G* + *jωC* (3)

where *G* is the conductance and *C* is the capacitance. Therefore, the muscle’s electrical properties, namely conductivity and relative permittivity, depend on conductance, capacitance and the geometric factor. Here, the conductivity is σ = *kG* and the relative permittivity is *ϵr = kC/ԑ*_0_. *k* is a proportional constant.

The FEM model developed and used in this study was designed based on the cross-section of the human upper arm. In this simulation, the magnetic induction effects are negligible and the field is considered quasi-static. Conservation of charge requires that:

(4) ∇·J=0

where *J* is the total current density. Thus, the equation that needs to be solved for the potential measurement *E* is:

(5) $$\nabla \cdot J_{total}=\nabla \cdot \left(\sigma E+j\omega \varepsilon_{0}\varepsilon_{r}E\right)=0$$

**Figure 1:**
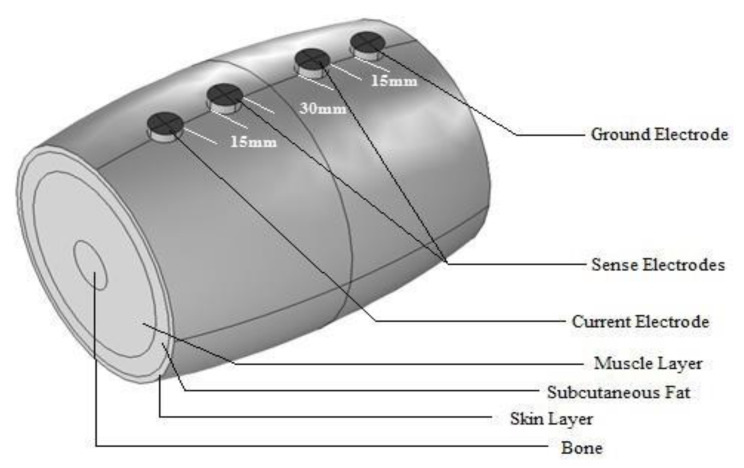
The FEM model of the human upper arm using COMSOL Multiphysics 4.2a (elbow to axilla), based on anatomic data. The inter-electrode spacing was 15 mm–30 mm–15 mm (60 mm in total).

In order to incorporate these basic equations in the frequency-dependent study, we have used COMSOL Multiphysics 4.2a as our simulation tool. The model comprises four different layers, i.e., skin, subcutaneous fat, muscle and bone marrow. All regions except muscle were assumed to be isotropic in nature, whereas muscle is anisotropic [16]. In practice, the varying concentrations of the intracellular and extracellular fluids alongside the muscle membranes affect the lipid bilayers of muscles, in effect, acting as an additional capacitor. However, to keep the model simple, these conditions were omitted. A 1 mA test current was supplied between the two current electrodes. For non-electrode boundaries, the normal component of the electric current was assumed to be continuous [17]. Electrodes were modelled as potential surfaces, the boundaries of which had either the excitation or the zero current, with the exception of the ground electrode, the potential of which was fixed at zero volts [15]. The discretization mesh was generated automatically with the COMSOL 4.3 software. Longitudinal and transverse conductivities and permittivities were obtained from the rat studies for 50 kHz [18] and were incorporated into the model, with rat data substituting for normal human muscle data. Fat and cortical bone marrow electrical data were obtained from Gabriel’s dielectric survey [19]. Our previous simulation showed that the placement of the bone inside the muscle does not affect the measured impedance. The rectangular electrode dimensions were 22 mm × 7 mm, and for the circular electrode the radius was 7 mm. For both the rectangular and circular electrodes, the total skin area covered by the electrodes was kept same, at 154 mm^2^. For this study, the inter-electrode distances (edge to edge) for both the circular and rectangular shapes were kept at 15 mm–30 mm–15 mm. The sensing electrodes’ voltage patterns were studied over a frequency range of 10 kHz to 1 MHz. Figure 1 depicts the FEM model designed for this study, with four different layers, along with electrodes.

**Figure 2:**
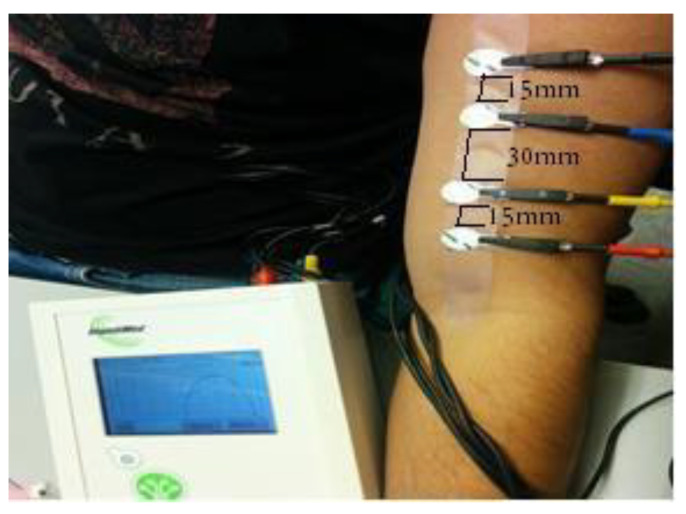
The EIM experiment setup on a human subject, with measurements shown.

For experimental verification, the study was conducted on 18 healthy human subjects. All procedures and methods were ultimately reviewed by the Georgia Southern University Institutional Review Board (IRB Protocol: H15367). The volunteers had no history of neuromuscular diseases. SFB7 from ImpediMed Inc. (Carlsbad, CA, USA) was used for measuring bioimpedance. This is a popular bioimpedance measuring instrument which can operate from 3 kHz to 1 MHz for a total of 256 discrete frequencies. A four-electrode setup in a parallel arrangement was used where the outer two electrodes delivered current at different frequencies, and the inner two electrodes measured the resulting voltage. Each 23 × 25 mm electrode strip (Part No. 292-STE; ImpediMed, Inc., [20]) was resized down to a length of 22 × 7 mm for the rectangular-shaped electrodes, and a circle with 7 mm radius for the circular-shaped electrodes, with a spacing of 10 mm–30 mm–15 mm between each strip (Figure 2). The gel supplied with the electrodes covered the entire area of an electrode. A paper model with the electrode configuration was designed to guarantee the reproducibility of the data collection [21]. Subjects were placed in a rest position at room temperature when EIM was measured. The arm was kept straight, and data collection took only a few seconds, without disturbing any cables connected with the analyzer.

**Figure 3:**
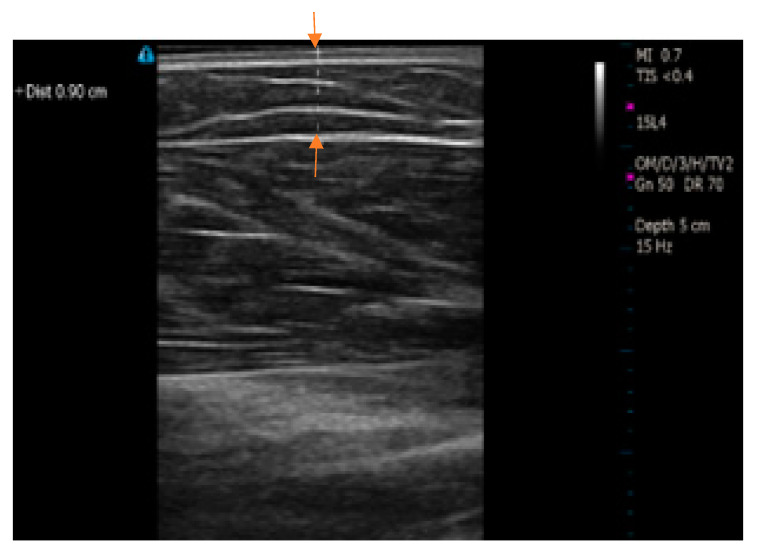
Ultrasound imaging for the measurement of subcutaneous fat thickness. Arrows show the measured skin-fat thickness.

Figure 3 illustrates the process of ultrasound imaging for measuring the fat thickness of individual subjects. The Terason t3200 B-Mode Portable Ultrasound (Terason, Burlington, MA, USA) was utilized to estimate skin-fat thickness. The ultrasound imaging was only applied to that area of the arm that was previously covered by the electrode setup while measuring the skin-fat thickness of the subject. In practice, SF thickness is not uniform along the length of the arm. In order to compensate for the variability, two measurements were taken at the two far ends, one at the approximate middle of the desired zone, and the average of these three measurements was recorded as the SF thickness for the individual subjects in this study.

## 3. Results

**Figure 4:**
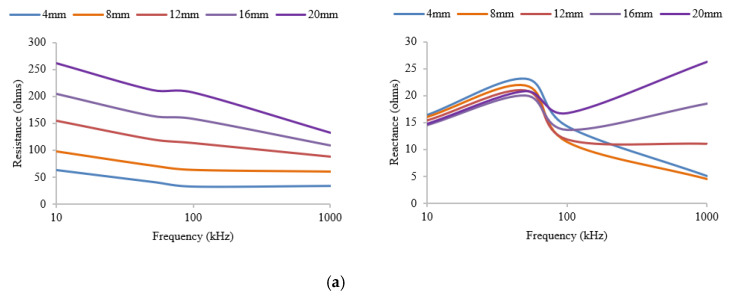

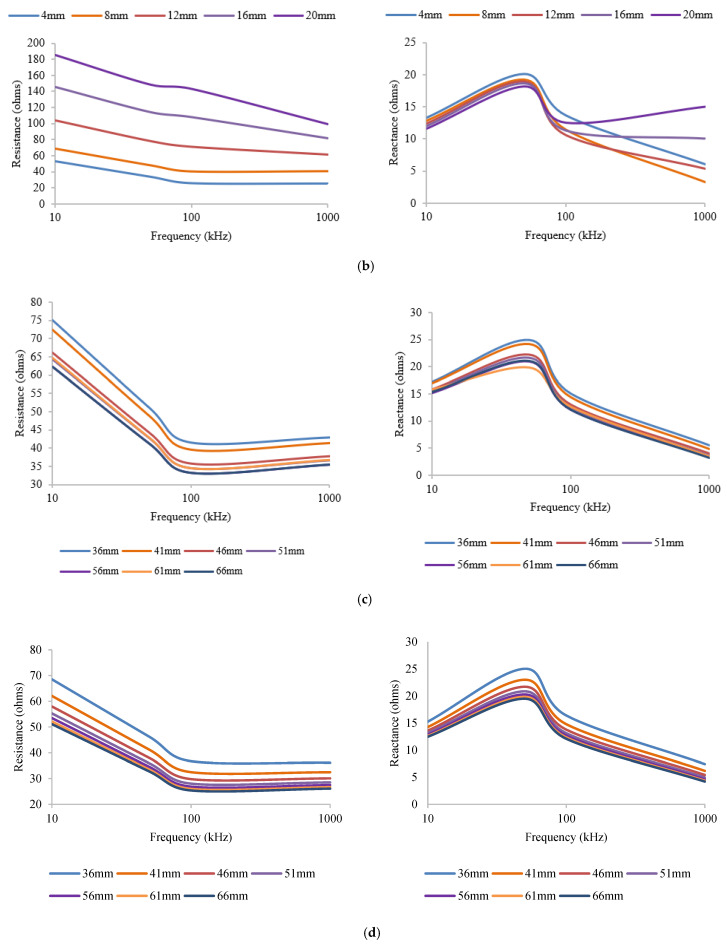
(**a**) Variations in EIM parameters with a 22 mm × 7 mm rectangular electrode, arranged with a 15 mm–30 mm–15 mm separation, for different SF thicknesses, with 52 mm muscle thickness. (**b**) Variation in EIM parameters with a 7 mm radius circular electrode, arranged with a 15 mm–30 mm–15 mm separation, for different SF thicknesses, with 52 mm muscle thickness. (**c**) Variation in EIM parameters with a 22 mm × 7 mm rectangular electrode, arranged with a 15 mm–30 mm–15 mm separation, for different muscle thicknesses, with 5 mm fat thickness. (**d**) Variation in EIM parameters with a 7 mm radius circular electrode, arranged with a 15 mm–30 mm–15 mm separation, for different muscle thicknesses, with 5 mm fat thickness.

Figure 4 illustrates the results of the effects of the anatomical factors we are considering in this study on each electrode shape. These results are based on the FEM study, and it is evident from these figures that the EIM parameters for both the rectangular and circular electrode shapes change in a similar manner over the frequency spectrum, as SF thickness varies. An earlier study [22] has shown that the real part of the impedance, i.e., resistance, is highly affected by the electrical properties of the skin-fat layer, whereas reactance, which is the imaginary part of the impedance, highlights changes in the thickness or electrical properties of the muscle. The isotropic skin-fat layer acts more like an electrical barrier, which increases the overall cross-sectional area of the muscle and thus increases the resistance. On the other hand, due to the anisotropic properties of muscle fibers, variations in the thickness of subcutaneous fat do not affect the reactance very much. However, in order to find out which electrode shape delivers a consistent profile, we have used linear regression analysis to find the smallest change in parameters per mm of thickness variation. Here, we have assumed that there is a linear relationship between changes in thickness and variations in the parameters. The electrode shape that demonstrates the minimum slope will be more acceptable than the other.

**Figure 5:**
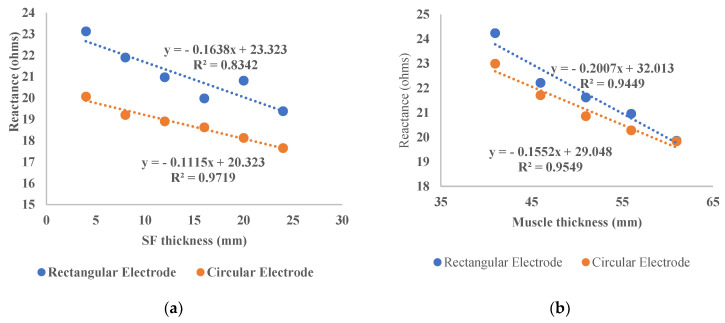
(**a**) Linear regression comparison between circular and rectangular electrodes for different subcutaneous SF thicknesses. (**b**) Linear regression comparison between circular and rectangular electrodes for different muscle thicknesses.

As can be seen from the above Figure 4a,b, for resistance, there is a strong dependence on subcutaneous fat thickness across the frequency spectrum. However, for both electrode shapes, reactance appears to be less affected, demonstrating considerably smaller changes within the frequency range of interest (10 kHz to 100 kHz). In the case of higher frequencies, EIM parameters show inconsistent behaviors. When the frequency increases, the current may begin to penetrate the sarcolemma and pass through the fibers directly [23]. At these higher frequencies, the proportion of intracellular water may start to play a more important role. At low frequencies, such as 10 kHz, both the electrode shapes display almost the same standard deviation of 0.48 ohms. However, in the case of 100 kHz, the reactance measured by the rectangular-shaped electrode has a standard deviation of 2.04 ohms, whereas that of the circular-shaped electrode is only 0.65 ohms. In addition, at higher frequencies, there is a 0.5-ohms increase in reactance per mm increase in fat thickness for the rectangular-shaped electrode, and only an increase of 0.14 ohms for the circular-shaped electrode. This provides evidence that EIM measurement by the circular electrodes is more effective as SF thickness varies. Figure 5a,b illustrate the advantage of circular electrodes over rectangular electrodes using a more statistical approach. For changes in both muscle and fat thickness, the reactance at 50 kHz has a smaller slope in the case of circular electrodes, thus demonstrating a decreased effect due to the changes in anatomical factors.

The measured subcutaneous fat thicknesses for eight subjects are listed in Table 1. Measurements show that the experiments covered a wide range of SF thicknesses, from 4 mm to 16 mm skin-fat thickness. In order to minimize the error in the experimental procedure, EIM measurements were conducted on both arms and the average of the measurements was analyzed for results. For single-frequency analysis, previous studies show that reactance at a certain frequency (e.g., 50 kHz) within the lower range of the spectrum is a suitable parameter for differentiating between healthy and unhealthy muscle [7,22]. Since the frequency at which the peak reactance occurs varies significantly between individuals, we have chosen a generic frequency (50 kHz) at which to compare the effectiveness of the two electrode shapes at varying thicknesses of subcutaneous fat. 

**Figure 6:**
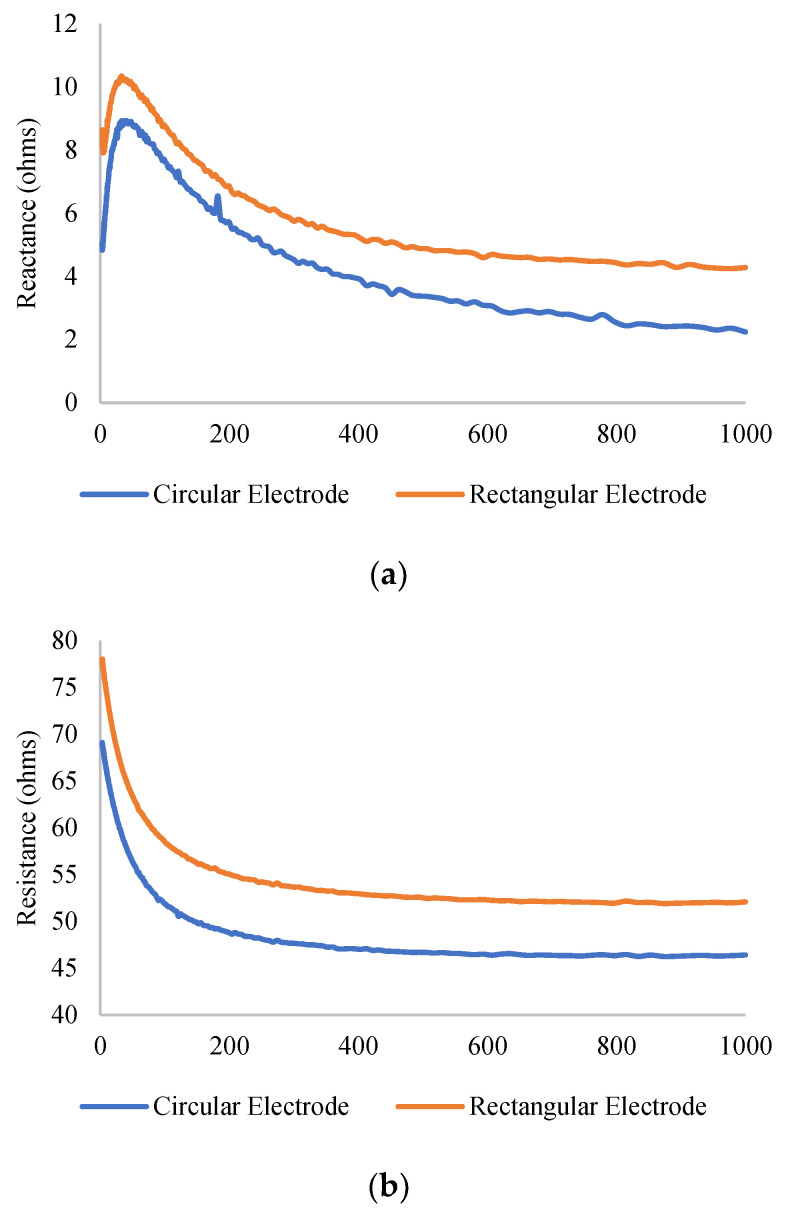
(**a**) The reactance plot for S3, with 9.8 mm skin fat. (**b**) The resistance plot for S3, with 9.8 mm skin fat.

**Figure 7:**
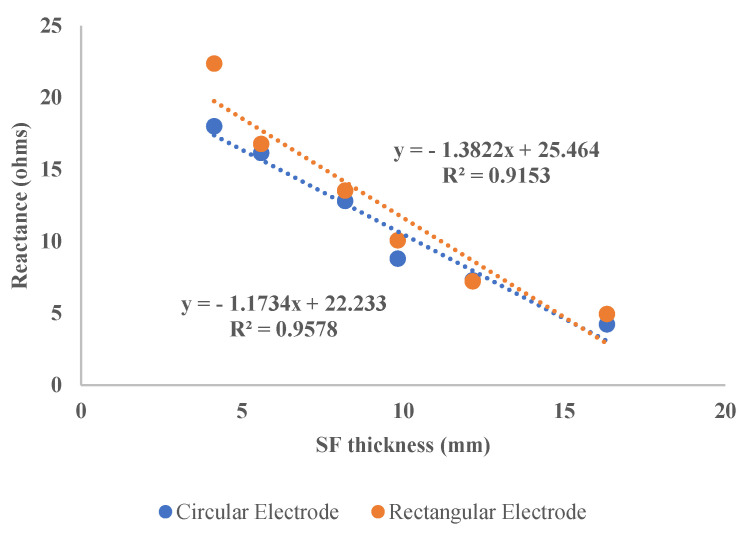
Linear regression comparison of the two electrode shapes.

The EIM experiments conducted on the human subjects proved the viability of our FEM study. For example, Figure 6a,b display similar changes in resistance and reactance for subject 3 (9.9 mm SF), for both electrode shapes. As predicted by our FEM model, the base impedance for the circular electrode is less than that of the rectangular electrode for the same subject. For a comparison of the two electrode shapes, Figure 7 illustrates the difference in slope with skin-fat thickness variation. To prove that there is a significant difference between the reactance values, we have conducted a one-tailed paired *t*-test on both reactance arrays, which resulted in a p value of 0.02518, meaning a significant difference exists between the two arrays.

It is evident from Figure 7 that the variation in reactance measured by circular electrodes with respect to variations in SF thickness is less than the variation in reactance obtained using rectangular electrodes. In addition, the EIM parameters obtained with circular electrodes are much more consistent, as can be observed from the R^2^ value. However, the values differ from the simulation results, as it was not practically possible to obtain subjects with varying SF thicknesses while keeping the muscle girth constant. 

**Figure 8:**
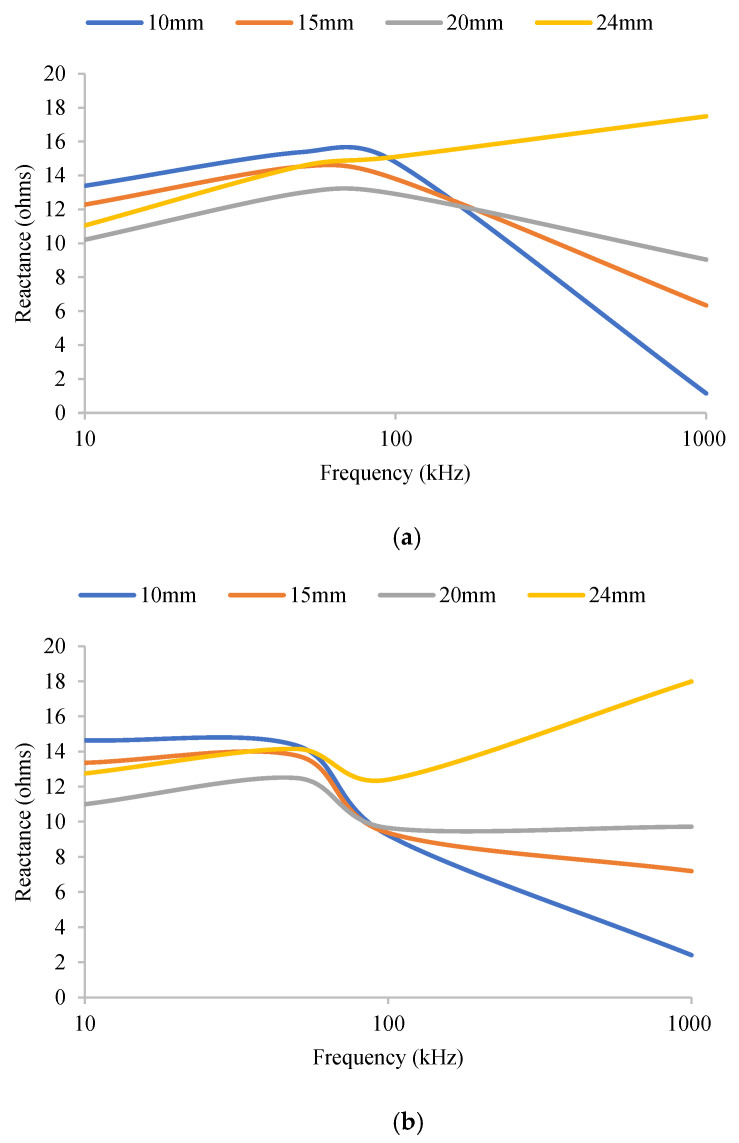
(**a**) FEM-predicted EIM properties in acute crush conditions for various SF thickness. (**b**) FEM-predicted EIM properties in chronic crush conditions, with circular electrodes for various SF thickness.

Though only skin-fat thickness was considered in this study, in practical scenarios both the muscle girth and skin-fat thickness vary between individuals. We have also incorporated rat muscle properties into the model, as stated in our methodology. The above discussion highlights that muscle thickness variation affects reactance more than all other EIM parameters. Despite the consideration that muscle thickness may increase or decrease with fat thickness, the circular electrode would be a better alternative to rectangular electrodes. Since the main goal of EIM is to detect neuromuscular disorders, we have extended our study to observe the effectiveness of circular electrodes in muscle with neuromuscular disease. For this purpose, acute and chronic crush data from a rat study [18] was incorporated into our FEM model. As can be observed from Figure 8a,b, circular electrodes can successfully distinguish acute and chronic crush conditions from normal muscle conditions.

## 4. Discussion

**Figure 9:**
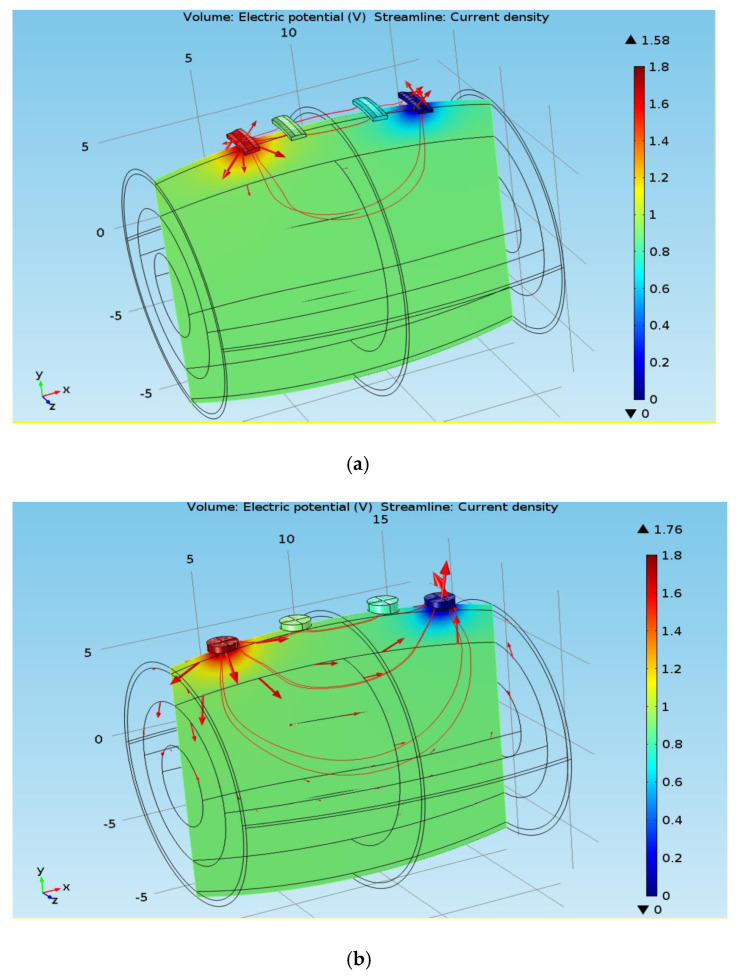
(**a**) Current distribution for rectangular electrodes with 17.4 mm SF thickness. (**b**) Current distribution for circular electrodes with 17.4 mm SF thickness.

The performance of the two different electrodes can be explained with the current distribution. The rectangular electrodes cover a larger area than the circular electrodes. Despite having the same surface area, the rectangular electrodes have a larger perimeter than the circular electrodes [21]. As a result, the injected current vectors are closer to each other in the case of circular electrodes, which forces the current components of the circular electrodes to be injected more perpendicularly to the plane than the rectangular electrodes (Figure 9). Due to the isotropic properties of skin and subcutaneous fat, a comparatively smaller amount of current is dissipated across both the layers due to the relatively perpendicular injection of the current. This causes the muscle fibers to be excited more in the case of circular electrodes, irrespective of variations in the thickness of the subcutaneous fat. However, the injected current component in the case of rectangular electrodes is more horizontally inclined, so that a considerable amount of the excitation current is exploited by the skin and the SF layer. For this reason, rectangular electrodes are more affected by changes in SF thickness.

Muscle is anisotropic, meaning it tends to allow more current to flow along the fibers rather than across the fibers, or in a direction that is transverse to the muscle bulk. Thus, both longitudinal and transverse conductivities and permittivities affect the EIM measurements. Earlier studies suggested that muscle injury tends to alter the longitudinal conductivity of muscle, which happens to be accentuated by reactance [24]. Therefore, larger excitation currents through the muscle fibers will result in decreased dependency on other anatomical factors and better determination of the muscle condition associated with the change in reactance. The geometric advantage of circular electrodes results in a concentrated current distribution in EIM experiments, relative to rectangular electrodes, which helps to decrease the effect on EIM parameters of subcutaneous fat thickness to a certain extent.

## 5. Conclusions

There are many challenges that exist in detecting neuromuscular diseases and monitoring the progress of diseases, since neurologists depend primarily on EMG data which is not always reliable, as discussed earlier. There is a tremendous need for alternative techniques that are reliable, noninvasive and cost-effective. EIM has proven its effectiveness thus far, but challenges remain before it can become a standardized tool for clinical purposes. The purpose and motivation for this study was driven by the fact that appropriate changes to the geometry of physical phenomena can alter the outcomes of a practical event. This study itself has produced several observations during the FEM simulations. One of the major EIM parameters, resistance, depends mostly on the dielectric properties of skin and SF tissue, whereas reactance is highly dependent on the dielectric properties of muscle. As a disease-diagnosis tool, EIM has great potential to escalate neuromuscular disease detection to a new level due to its accuracy and noninvasive nature. However, it is hindered by its unavoidable dependency on other anatomical and non-anatomical factors. The use of circular electrodes instead of rectangular electrodes could help overcome the dependency of EIM parameters to some extent, as demonstrated by our FEM study and practical experiments. However, the electrode separation also affects EIM parameters, and our future goal is to propose an optimized electrode configuration setup that will reduce the variation of EIM parameters to a minimum level. In this study, although multifrequency data was collected over the range of 3 kHz to 1 MHz, only data at 50 kHz were analyzed, for reasons described earlier. However, multifrequency data analysis can also provide rich insights into bioimpedance measurement, which we plan to perform in the future. Another limitation of EIM is that there is no bioimpedance analyzer that safely measures impedances in humans and thus there is tremendous need for an analyzer that can measure bioimpedances up to the GHz range. The results found in this study will help EIM to be used effectively for monitoring the progression or recession of neuromuscular diseases in clinical settings, even for patients with obesity. 

## Figures and Tables

**Table 1:** Measured SF thickness of the eight individual subjects.

Subject ID	M1 (cm)	M2 (cm)	M3 (cm)	Avg (cm)	SD (cm)
S1	1.20	1.23	1.22	1.2166667	0.02
S2	1.62	1.63	1.65	1.6333333	0.02
S3	0.99	0.98	0.98	0.9833333	0.01
S4	0.90	0.89	0.90	0.8966667	0.01
S5	0.78	0.81	0.83	0.82	0.03
S6	0.42	0.42	0.40	0.4133333	0.01
S7	0.60	0.57	0.59	0.5866667	0.02
S8	0.55	0.55	0.58	0.56	0.02

## Data Availability

All simulation data are presented in this paper. Experimental data presented here can be requested from the corresponding author. This data are not publicly available due to the privacy.
